# Does ambient voice technology in healthcare really save time? The need for rigorous evaluation to test the promise

**DOI:** 10.1136/bmjdh-2026-000039

**Published:** 2026-03-18

**Authors:** Niels Peek, Matthew Woodward, Lukasz Zielinski, Ross Goldstone, Mary Dixon-Woods

**Affiliations:** 1 THIS Institute, Department of Public Health and Primary Care, University of Cambridge, Cambridge, UK; 2 The Health Foundation, London, UK; 3 Barts Health NHS Trust, London, UK

**Keywords:** Artificial intelligence, Documentation, Outcome and Process Assessment, Health Care

## Introduction

Bold promises are currently being made for how artificial intelligence (AI) could transform healthcare. Examples include AI-enabled booking systems, retrieving and organising patient records, and increasingly, ambient voice technology (AVT). AVT systems (also called ‘digital scribes’ or ‘clinical AI scribes’) passively capture clinician–patient conversations and automatically generate clinical documentation. The rapid emergence of AVT suppliers, growth in professional interest and enthusiasm at policy level—as reflected in the UK government’s 10-Year Health Plan^1^—is already driving fast adoption. Much of the uptake is based on promises of increased productivity and better patient and staff experience linked to freeing up professional time. Critical questions arise about whether AVT claims are well-founded.

The scientific evidence base is still emerging, often relying on weak study designs. Studies show variable and conflicting findings: one US study reported that approximately half of participants achieved decreased documentation time and reduced after-hours work,^2^ whereas another found 20%–30% time savings (though benefits varied widely between clinicians).^3 4^ The first randomised controlled trial of AVT, published in late 2025, found that one AI scribe application reduced note-writing time by approximately 10%, with modest improvements in physician burnout measures, whereas a second showed no benefit.^5^

## Addressing the evidence gap

Professional time is among the most valuable assets in healthcare but is also one of the least well studied. For example, ‘releasing time to care’,^6^ a productivity initiative implemented in 70% of acute wards in England from 2008 onwards, lacked robust evaluation of whether time was actually released.

Measuring how healthcare staff spend their time is methodologically complex. Self-reported time-use logs are prone to recall bias and may not reflect actual behaviour. Logs of interactions with clinical systems typically fail to capture multitasking, interruptions and offline activities. Time-and-motion studies, although more objective, are resource-intensive^7^ and can struggle to capture the fragmented, multitasking nature of healthcare work. For AVT, clinician supervision is necessary to ensure accuracy and completeness of documentation, so time required for output checking could easily erode any marginal gains. Uncorrected errors may displace workload elsewhere, or even lead to clinical negligence claims.^8^ Studies to date have mostly focused on the role of the technology in improving efficiency and productivity, not patient outcomes. While AVT tools are not currently involved in clinical decision-making per se, they interact closely with clinical workflows and could impact patient outcomes in ways not yet fully understood or systematically managed.

Despite its promise to liberate time into more patient-facing care or rest for overburdened staff, AVT might instead shift time use to different tasks, forms of documentation or interaction with patients and colleagues.^9^ Whether this leads to improvements in care quality, clinician well-being or system efficiency remains unanswered. A shift from manual to automated documentation may impact clinicians’ ability to structure their thoughts, reflect and remember and accordingly on training and service quality.

A further methodological challenge arises from variability in AVT products themselves. The offerings of the numerous vendors in the market differ not only in their core transcription and summarisation capabilities but also in adjacent features such as coding support, letter generation and integration with electronic health records. Moreover, these products evolve rapidly, often updating their underlying models and functionality between studies, making it difficult to establish a stable evidence base or to generalise findings from one product to another.

Models for regulation and governance also remain to be settled. An NHS England priority notification in June 2025 warned that many AVT solutions then in use did not meet required standards and directed pausing of engagement with non-compliant suppliers. In other jurisdictions, AVT may not be regulated as a medical device at all.

In summary, evaluating the real impact of AVT requires careful attention not only to quantitative measures of time saved but also to qualitative assessments of how that time is experienced and repurposed and the proximal and distal effects of their deployment. Without this, claims of productivity and efficiency gains risk being speculative, driven by interests or misrepresentative of the pressures and complexities of clinical work. Addressing the evidence gaps is therefore essential.

## Looking ahead

AVT promises to reshape healthcare documentation—moving from manual entry to real-time, AI-generated summaries embedded seamlessly into clinical encounters. If implemented thoughtfully, this shift has the potential to significantly reduce administrative burdens, improve documentation quality and timeliness and allow clinicians to focus more fully on patient care.

However, we need to get this right. Careful stewardship is required, with developers, researchers, policymakers and regulators working collaboratively. Robust evaluation needs to be a non-negotiable requirement of deployment while centring staff and patient perspectives in design and governance through robust co-design,^10^ and developing regulatory frameworks that reflect this technology’s distinctive properties. Evaluation should combine quantitative measurement of time use (ideally using time-and-motion methods or validated system log analysis) along with qualitative investigation, including downstream effects on documentation quality, error rates, clinician satisfaction and patient outcomes, using controlled designs with adequate follow-up periods.

## Data Availability

Data sharing not applicable as no datasets generated and/or analysed for this study.
